# The wellbeing gap between immigrants and natives: the role of social integration

**DOI:** 10.3389/fsoc.2026.1815828

**Published:** 2026-06-19

**Authors:** Fari Aftab, Juliane Scheffel, David Spencer

**Affiliations:** 1 Cardiff Business School, Cardiff University, Cardiff, United Kingdom; 2 Leeds University Business School, Leeds, United Kingdom

**Keywords:** British identity, immigration, mental health, neighborhood attachment, social integration, social networks, subjective wellbeing, UKHLS

## Abstract

**Introduction:**

Existing research has examined immigrants' subjective wellbeing and economic integration but less is known about whether multiple dimensions of social integration jointly account for differences in subjective wellbeing between immigrants and natives in the UK. This paper examines whether British identity, community involvement, neighborhood attachment and UK-based social networks help explain this disparity.

**Methods:**

The analysis uses waves 3 and 6 of the UK Household Longitudinal Study and estimates correlated random effects models for two outcomes: life satisfaction and GHQ-based mental health. The models first estimate the immigrant-native gap, then add demographic characteristics and economic integration controls, before introducing the selected social integration measures.

**Results:**

Immigrants report lower life satisfaction and mental health than natives, and these differences remain after demographic and economic integration controls are included. Adding social integration measures substantially reduces the remaining disparity. British identity, neighborhood attachment and UK-based social networks have the clearest explanatory relevance, while community involvement plays a more limited role.

**Discussion:**

The findings suggest that differences in subjective wellbeing between immigrants and natives in the UK are associated not only with economic integration, but also with social connection, local embeddedness and subjective belonging. Integration should therefore be understood as a multidimensional process, although the results are associational and should not be interpreted causally.

## 1 Introduction

The integration of immigrants in host societies remains a central issue for public policy and sociological research, with implications for subjective wellbeing, equality of opportunity and social cohesion (Ager and Strang, 2008; Demireva, 2019; OECD and European Commission, 2023; Šedovič, 2024). This is especially relevant in the UK, where a long and diverse migration history intersects with continuing debates about British identity, multiculturalism, local belonging and community relations (Cook et al., 2011; Saggar et al., 2012; Heath and Demireva, 2014; Jaspal et al., 2021). Existing evidence shows that immigrants often report lower subjective wellbeing than natives, although the size and direction of this gap vary across national contexts, immigrant groups and wellbeing outcomes (Safi, 2010; Bartram, 2011; Kirmanoglu and Başlevent, 2014; Arpino and de Valk, 2018; Tegegne and Glanville, 2019). Against this background, this paper examines the immigrant-native subjective wellbeing gap in the UK and asks whether multiple dimensions of social integration help account for that disparity.

The study of this gap matters for both academic and policy reasons. Subjective wellbeing captures how individuals evaluate their lives, circumstances and opportunities (Paparusso, 2021). For immigrants, lower wellbeing may reflect not only economic disadvantage, but also discrimination, social isolation, weaker social support, limited social capital and uncertainty about belonging in the host society (Safi, 2010; De Vroome and Hooghe, 2014; Kirmanoglu and Başlevent, 2014; Tegegne and Glanville, 2019). Integration is therefore understood here not only in terms of structural outcomes such as employment and income, but also through participation, social relationships, local embeddedness and belonging (Ager and Strang, 2008; Saggar et al., 2012; Zafar and Ammara, 2023). In this paper, integration is examined comparatively, as the extent to which immigrants and natives differ across the economic and social dimensions under investigation.

A substantial strand of migration research examines integration through economic outcomes such as education, employment, income and home ownership. These factors remain important because they shape material security, occupational opportunities, social status and settlement prospects (Dustmann, 1996; Sinning, 2010; Kóczán, 2016; Gobillon and Solignac, 2020). Income and employment are particularly relevant to wellbeing: Bartram (2011) shows that income is more strongly associated with happiness among immigrants than among natives in the United States, while (Kóczán 2016) finds that employment accounts for an important part of immigrants' lower life satisfaction in Germany. Wider evidence also shows that unemployment has substantial wellbeing consequences, including among immigrant populations (McKee-Ryan et al., 2005; Leopold et al., 2017; Shen and Kogan, 2020). Home ownership and education also matter because they reflect settlement, economic security and access to opportunity (Schellenberg and Hou, 2005; Constant et al., 2009; Sinning, 2010; Mazzucato et al., 2017; Zumbro, 2014; Lai et al., 2021; Son and Choi, 2026). These economic factors are therefore included as controls against which the additional explanatory role of social integration can be assessed.

Moving beyond economic outcomes, a growing body of evidence shows that immigrant subjective wellbeing is shaped by social capital, social embeddedness, neighborhood context, identity and exclusion. Safi (2010) shows that immigrants' life satisfaction in Europe is shaped by both assimilation and discrimination, while (Kirmanoglu and Başlevent 2014) show that immigration, discrimination and citizenship condition life satisfaction among ethnic minority members. Tegegne and Glanville (2019) find that lower social capital explains a substantial part of the non-western immigrant-native subjective wellbeing gap across Western Europe. Ambrosetti and Paparusso (2024) show that both first- and second-generation immigrants report lower life satisfaction than natives, and that perceived neighborhood quality is positively associated with life satisfaction. These studies are directly relevant to the present paper, but they also indicate that social integration cannot be reduced to a single measure of social capital. The present analysis therefore examines four observable social dimensions together: British identity, community involvement, neighborhood attachment and UK-based social networks.

British identity captures symbolic attachment to the host society and a subjective sense of belonging within the national community. This is especially relevant in the UK, where Britishness has been closely connected to debates about migration, multiculturalism and minority integration (Manning and Roy, 2010; Heath and Demireva, 2014; Liu, 2020; Jaspal et al., 2021). Manning and Roy (2010) show that immigrants are less likely than UK-born respondents to report a British identity on arrival but become more likely to do so with longer residence. Casey and Dustmann (2009) also show that identity is associated with economic and intergenerational outcomes, while Angelini et al. (2015) link cultural assimilation to immigrants' life satisfaction. UK evidence further shows that British national identification is positively associated with life satisfaction among ethnic minority respondents (Jaspal et al., 2021). In this paper, British identity is treated as an indicator of host society attachment, not as a requirement that immigrants abandon origin identities.

Community involvement captures active engagement through volunteering and charitable behavior. These forms of participation are often treated as indicators of civic and social integration, although they do not all measure the same mechanism (Osili and Du, 2005; Handy and Greenspan, 2009; Greenspan et al., 2018). Volunteering can support social contact and signal socio-cultural participation (Baert and Vujić, 2016; Greenspan et al., 2018), while charitable behavior may reflect broader incorporation into host society patterns of giving (Osili and Du, 2005). The wider wellbeing literature links volunteering and giving to altruism, recognition and warm-glow benefits (Andreoni, 1990; Appau et al., 2019). Evidence from immigrant specific studies is mixed: Ramos et al. (2017) find that community involvement is positively associated with life satisfaction among Hispanic immigrants in rural Nebraska, while Hombrados-Mendieta et al. (2013) show that sense of community is associated with higher life satisfaction in Spain. However, Miller et al. (2020) find that formal volunteering is not significantly associated with migrant mental wellbeing in Tokyo after adjustment. This mixed evidence supports examining community involvement alongside other, more relational dimensions of integration.

Neighborhood attachment captures everyday interaction, local embeddedness and connection to the immediate residential area. Neighborhoods can provide opportunities for social contact, practical support, trust and local belonging (Cook et al., 2011; Knies et al., 2016). Appau et al. (2019) provide UK evidence that neighborhood belonging, and interaction are associated with life satisfaction in the general population. Knies et al. (2016), using Understanding Society, show that ethnic minorities in England report lower life satisfaction than the White UK majority and that own-group concentration can be positively associated with life satisfaction for some minority groups. Ambrosetti and Paparusso (2024) further show, using European data, that subjective evaluations of neighborhood characteristics and services are positively associated with life satisfaction among both immigrants and natives. Neighborhood attachment is therefore included as a distinct dimension of social integration that captures place-based belonging and local embeddedness.

Social networks provide a broader measure of interpersonal connection beyond the immediate neighborhood. Close UK-based friendships may reduce isolation and provide emotional, practical and informational support. This is consistent with evidence that social contact and close personal ties explain part of the immigrant-native wellbeing gap across Europe (Arpino and de Valk, 2018), and that informal social connections and trust explain a substantial share of the non-western immigrant-native subjective wellbeing gap (Tegegne and Glanville, 2019). Research on migration and settlement also shows that social ties, information networks and close relationships shape migrant outcomes (Korinek et al., 2005; Danzer and Ulku, 2011; Meng and Xue, 2020). Qualitative UK evidence further shows that migrant settlement is supported by a range of local relations, including acquaintances and friendships with co-ethnics, other migrants, ethnic minority British people and members of the White British majority (Wessendorf and Phillimore, 2019). The present measure of UK-based close friendships is therefore used as a transparent, though necessarily limited, proxy for host country social embeddedness.

These social dimensions may be relevant not only to life satisfaction but also to mental health. Brydsten et al. (2019) show that social integration, social support, trust and economic insecurity help explain mental health inequalities between foreign-born and native-born people in Sweden. Broader evidence on immigrant mental health also points to the role of post-migration conditions, stressors, social support and settlement context (Jayaweera, 2014; Gilliver et al., 2014; Hou et al., 2020). The present paper therefore uses both life satisfaction and GHQ-based mental health as outcomes. This allows us to examine whether the explanatory relevance of social integration is similar across evaluative and psychological dimensions of subjective wellbeing.

The four social integration measures used here are theoretically grounded but not exhaustive. Religious involvement, discrimination, legal status, migration history, origin country conditions and ethnic composition of networks may also shape immigrant wellbeing. For example, Tegegne and Glanville (2019) note that immigrants may have higher religious involvement while still having lower overall social capital than natives. These dimensions are not ignored conceptually, but they are beyond the direct empirical scope of the present paper. The contribution is therefore not to claim that British identity, community involvement, neighborhood attachment and UK-based social networks capture all aspects of integration. Rather, they provide observed UKHLS indicators of symbolic belonging, civic engagement, local embeddedness and close interpersonal ties, allowing these dimensions to be examined jointly within the same immigrant-native panel framework.

The UK provides an important setting for this analysis. It has a long and diverse migration history shaped by postcolonial, EU, refugee and more recent non-EU migration, as well as continuing public debates about British identity, multiculturalism, social cohesion and local belonging (Manning and Roy, 2010; Saggar et al., 2012; Heath and Demireva, 2014). German panel studies and European comparative studies provide important evidence on immigrant wellbeing, identity, social capital and labor market conditions (Safi, 2010; Casey and Dustmann, 2009; Angelini et al., 2015; Kóczán, 2016; Arpino and de Valk, 2018; Tegegne and Glanville, 2019; Ambrosetti and Paparusso, 2024). However, these findings cannot be assumed to fully explain how British identity, local attachment and UK-based friendships relate to immigrant-native wellbeing differences in the UK. UKHLS is well suited to this purpose because it provides repeated measures of subjective wellbeing, demographic and economic circumstances, and several dimensions of social integration within the same national panel.

This paper contributes to the literature in three ways. First, it provides UK-specific panel evidence on the immigrant-native subjective wellbeing gap, adding national depth to a literature that includes important European comparative studies and German panel evidence. Second, it treats social integration as a multidimensional explanatory framework by examining British identity, community involvement, neighborhood attachment and UK-based social networks jointly rather than as isolated indicators. Third, it identifies which social dimensions account for the remaining wellbeing gap after demographic characteristics and economic integration factors are taken into account. This reframes the contribution: the paper does not claim that social integration has been neglected altogether but asks which dimensions of social integration carry the greatest explanatory relevance in the UK context.

The paper addresses three research questions. First, do immigrants in the UK report lower subjective wellbeing than natives after accounting for demographic characteristics and established indicators of economic integration? Second, to what extent do British identity, community involvement, neighborhood attachment and UK-based social networks help explain this wellbeing disparity? Third, are the findings consistent across life satisfaction and mental health? The analysis produces three main findings. Immigrants report lower subjective wellbeing than natives; social integration measures account for a substantial share of the remaining gap, especially British identity, neighborhood attachment and UK-based social networks; and the broad pattern is similar across life satisfaction and mental health, although the immigrant coefficient becomes statistically insignificant only in the fully specified mental health model.

The remainder of the paper is structured as follows. Section 2 presents the data and methodology, organized around the data source, research methodology and methods of analysis. Section 3 presents the results. Section 4 presents the discussion and conclusion.

## 2 Data and methodology

### 2.1 Data source

To explore the relationship between social integration of immigrants and subjective wellbeing in the UK, we use the United Kingdom Household Longitudinal Study (UKHLS), a nationally representative household and individual-level panel survey (University of Essex, Institute for Social and Economic Research, 2025). The survey began in 2009 and has approximately 40,000 participating households across England, Scotland, Wales, and Northern Ireland. The dataset is organized as 'waves', where each wave captures the responses for two to three overlapping years. Fortunately for our purposes, the dataset has a large general population sample, an ethnic minority booster sample, and an additional immigrant-ethnic minority boost sample (included from wave six), containing rich information on respondents' characteristics and behavior.

We use waves 3 (2011–2013) and 6 (2014–2016) for our analysis because these waves provide measures of wellbeing and social integration for the same individuals over time, thereby preserving the panel structure of the dataset. We classify immigrants as all non-UK born individuals and natives as UK-born individuals for ease of description and use. This definition is in line with previous studies (Casey and Dustmann, 2009; Sinning, 2010; Dustmann and Frattini, 2011).

#### 2.1.1 Indicators of subjective wellbeing

Our first outcome variable for subjective wellbeing is life satisfaction which captures people's self-reported assessments of the quality of their lives. This measure is widely supported in previous studies as valid and reliable (e.g., Dolan and Kahneman, 2008; Layard, 2011). In UKHLS, individuals are asked: “*How satisfied, or dissatisfied, are you with your life overall*?” Respondents can then answer this question on a Likert scale of 1 for completely dissatisfied to 7 for completely satisfied.

Our second outcome variable for subjective wellbeing is mental health, which is measured using the General Health Questionnaire (GHQ-12) and is considered a reliable and valid measure of mental wellbeing (Griffith and Jones, 2019). This measure is based on responses to 12 separate questions regarding respondents' ability to concentrate, overcome difficulties, make decisions, solve problems, enjoy everyday activities, sleeplessness, feelings of usefulness, strain, depression, confidence, self-worth, and happiness. In the UKHLS, the scale is originally constructed so that higher values indicate greater psychological distress. For ease of interpretation and consistency with life satisfaction, we reverse code this measure so that higher values indicate better mental health. The resulting scale ranges from 0 to 12, with higher scores representing better mental health.

#### 2.1.2 Indicators of social integration

To study whether various aspects of social integration help account for the subjective wellbeing disparity between immigrants and natives, we have carefully selected four social concepts. These are national identity, community involvement, neighborhood attachment and social networks within the UK. The proxy measures of these are discussed below.

#### 2.1.3 National identity

Our measure of national identity is based on a question asked in the UKHLS regarding the importance of being British and ranges from 0 to 10. Respondents were asked: *Most people who live in the UK may think of themselves as being British in some way. On a scale of 0 to 10 where 0 means ‘not at all important' and 10 means ‘extremely important', how important is being British to you?*

#### 2.1.4 Neighborhood attachment

The UKHLS dataset asks questions regarding respondents' attachment to the neighborhood. We generate a measure of neighborhood attachment based on the information from three different question statements, where respondents were asked to report their level of agreement/disagreement [scale from 1 (strongly disagree) to 5 (strongly agree)].

The question statements are: *(1) I regularly stop and talk with people in my neighborhood; (2) If I needed advice about something I could go to someone in my neighborhood; (3) I borrow things and exchange favors with my neighbors*. For interpretation and use, we summed up the responses to the three question statements, thus creating a scale measuring neighborhood attachment that ranges from 1 (low attachment) to 15 (high attachment). The three items show acceptable internal consistency, with Cronbach's alpha of 0.76. This supports their use as a combined measure of neighborhood attachment, capturing everyday interaction, access to advice and reciprocal exchange with neighbors.

#### 2.1.5 Social networks within the UK

We use social networks within the UK, other than family members as an additional indicator of social integration. This allows us to capture social integration in a broader sense, thus departing from a localized view of integration in the host country as in the case of neighborhood attachment. The variables used to generate a measure of social networks within the UK are based on the questions asked of respondents about the location of their three best friends: *How far away each friend lives: (1) Less than 1 mile, (2) less than 5 miles, (3) between 5 and 50 miles, (4) over 50 miles but still within the UK and (5) this friend lives in another country*.

We code the responses for each friend into two groups (thus, creating three dummies). ‘1′ is coded for those with friends that live in categories 1 to 4 and category 5 is coded ‘0′^1^. For interpretation and use, we summed up the three dummy variables to generate a final variable for social networks within the UK that ranges from ‘0′ for lack of a best friend in the UK to ‘3′ for having a maximum of at least three best friends located in the UK.

#### 2.1.6 Community involvement

We capture community involvement of individuals by volunteering and charitable behavior. In relation to volunteering, respondents are asked: “*In the last 12 months, have you given any unpaid help or worked as a volunteer for any type of local, national or international organization or charity?*”.

To capture charitable behavior, respondents are asked: “*In the last 12 months, have you donated any money to charities or other organizations?*” These questions are based on ‘Yes' and ‘No' responses and are used to construct dummy variables, which are equal to 1 for ‘Yes' for volunteering and charitable behavior and 0 otherwise.

#### 2.1.7 Data harmonization and robustness checks for community involvement

The empirical analysis uses waves 3 and 6 of the UKHLS because these waves provide the subjective wellbeing outcomes and the principal social integration measures for the same individuals over time. Most variables used in the analysis are observed in both of these waves. The exception is community involvement. The two variables used to capture this dimension, volunteering and charitable behavior, are observed in waves 4 and 6 rather than waves 3 and 6. This creates a data harmonization issue because excluding these variables would omit one dimension of social integration, while restricting the analysis only to waves in which all variables are observed would leave a single cross-section in wave 6 and remove the panel structure required for the correlated random effects specification.

The main analysis therefore uses the closest available observation for community involvement. Specifically, wave 4 values of volunteering and charitable behavior are assigned to the wave 3 record for the same respondent, while wave 6 values are used directly. This allows all dimensions of social integration to be examined jointly within a consistent two wave analytical sample. The procedure is a data harmonization strategy rather than a claim about temporal causality. We do not interpret volunteering or charitable behavior in wave 4 as causes of subjective wellbeing in wave 3. Instead, these variables are used to capture respondents' broad community involvement around the relevant period. The analysis remains associational, and the community involvement results are therefore interpreted cautiously.

We assess whether this decision affects the findings through three robustness checks. First, we re-estimate the main models excluding volunteering and charitable behavior from the social integration block. Second, we estimate wave 6 only cross-sectional models in which all social integration variables, including volunteering and charitable behavior, are directly observed. Across both checks, the results remain substantively consistent with the main analysis: the direction of the key associations is unchanged, and the conclusions regarding the explanatory relevance of the social integration variables remain the same. These results show that the central findings do not depend on the inclusion or harmonization of the community involvement variables, as reported in Appendix Tables A1–A4.

Third, we examine the stability of volunteering and charitable behavior across waves 4 and 6, where both variables are directly observed. As shown in Appendix Table A5, 82.9 percent of respondents remain in the same volunteering category and 75.8 percent remain in the same charitable behavior category across the two waves. The wave 4 to wave 6 correlations are 0.476 for volunteering and 0.392 for charitable behavior. Immigrant-native differences are also consistent across the two observed waves. For volunteering, the native mean is 0.207 in wave 4 and 0.213 in wave 6, compared with 0.174 and 0.189 among immigrants. For charitable behavior, the native mean is 0.712 in wave 4 and 0.750 in wave 6, compared with 0.641 and 0.685 among immigrants. These patterns indicate moderate persistence in the community involvement measures and suggest that the observed immigrant-native differences are not an artifact of the harmonization approach.

Taken together, these checks support the reliability of the empirical approach. The timing of volunteering and charitable behavior is a limitation, and the results for community involvement should not be interpreted as establishing temporal precedence. However, the robustness checks show that the main conclusions are not driven by the treatment of these variables. The principal explanatory pattern rests on British identity, neighborhood attachment and UK-based social networks, while community involvement has a more limited role in the panel specifications.

#### 2.1.8 Control variables

Demographic controls include a dummy for female *(female* = *1 and male* = *0)*, marital status *(unmarried* = *0, married* = *1, divorced* = *2)*, age and age squared, good health (*no long-term illness/disability* = *1, otherwise* = *0*) and a discrete variable for household size. Age squared is included to allow for a non-linear relationship between age and subjective wellbeing, as previous research often finds wellbeing to vary across the life course rather than changing linearly with age (Baltatescu, 2005; Safi, 2010; Arpino and de Valk, 2018; Appau et al., 2019).

Economic integration controls include a categorical variable for educational qualifications *(1* = *Degree/ higher degree, 2* = *no qualifications, 3* = *GCSE/Lower and 4* = *A-levels)*, employment status *(1* = *paid employment, 2* = *unemployed, 3* = *Outside the labor market*), a dummy for home ownership *(1*= *owned outright or owned on mortgage/shared ownership, 0* = *otherwise)*, and a continuous variable for logged net monthly household income.

### 2.2 Research methodology

Our analysis begins by examining how individuals' subjective wellbeing, measured by life satisfaction and mental health, is associated with immigrant status and indicators of social and economic integration. The aim is to assess whether immigrants report lower subjective wellbeing than natives, and whether this difference is reduced after accounting for demographic characteristics, economic integration and the selected dimensions of social integration. This yields the following model specification:


$$\begin{array}{c} Y_{it}=\;\alpha_{0}+\;\alpha_{1}immigrant_{i}+X_{it}\alpha_{2}+Z_{it}\alpha_{3}+\delta_{t}+\;\mu_{it}, \end{array}$$ (1)

where *Y*_*it*_ represents subjective wellbeing outcome for individual *i* at time *t*. In the main analysis *Y*_*it*_ is measured first by life satisfaction and then by mental health. The variable *immigrant*_*i*_ is a dummy variable identifying non-UK born individuals and enables us to estimate the difference in subjective wellbeing between immigrants and natives. **X**_**it**_ is a vector of demographic characteristics and indicators of economic integration. These include gender, marital status, age and age squared, household size, health, education, employment status, home ownership and logged net monthly household income. **Z**_**it**_ is a vector representing the selected indicators of social integration, namely British identity, community involvement, neighborhood attachment and social networks within the UK. δ_*t*_ denotes wave dummies, which account for period specific influences that may affect respondents' subjective wellbeing similarly across the sample. The composite error term μ_*it*_ consists of an unobserved individual component and an idiosyncratic error term.

### 2.3 Methods of analysis

The panel structure of UKHLS is important for the present analysis because it provides repeated observations for the same individuals. This enables the analysis to move beyond a single cross-sectional comparison and to use variation in respondents' characteristics and answers across waves. In practical terms, this is incorporated using wave indicators and a correlated random effects specification in which individual means of time-varying covariates are included following Mundlak (1978). The wave indicators capture differences across survey waves, while the inclusion of person specific means of time varying variables allows us to account for time-invariant unobserved individual heterogeneity that is correlated with observed time-varying covariates.

This approach is appropriate for the research questions addressed in this paper. A standard fixed effects model would control for all time-invariant individual characteristics, but it would also absorb immigrant status, since immigrant status is time-invariant in our analysis. This would prevent us from estimating the immigrant native subjective wellbeing gap directly. A standard random effects model, by contrast, would retain the immigrant coefficient, but would rely on the assumption that unobserved individual effects are uncorrelated with the explanatory variables. This assumption is unlikely to hold in the present context, because relatively stable individual characteristics, such as personality, reporting style, cultural background or long-standing orientations toward social life, may be associated both with subjective wellbeing and with some measures of social or economic integration.

The correlated random effects approach offers a suitable compromise. It retains the ability to estimate the coefficient on immigrant status while relaxing the restrictive assumption of the standard random effects model. Following Mundlak (1978), we include the individual means of time varying explanatory variables in the model. This allows the unobserved individual component to be correlated with the observed time-varying covariates. In this sense, the model uses the panel structure of the data to reduce bias arising from time-invariant unobserved heterogeneity, while preserving the central comparison between immigrants and natives.

Several model evaluation checks were undertaken to assess the suitability of the empirical specification. First, Breusch Pagan Lagrange Multiplier tests rejected pooled OLS in favor of a panel specification for both life satisfaction and mental health. Second, fixed effects and standard random effects models were estimated for comparison. The Hausman tests were statistically significant for both outcomes, indicating that the assumptions of standard random effects are restrictive. However, fixed effects models cannot provide the main specification for this paper because immigrant status is time-invariant and is therefore absorbed by individual fixed effects. The correlated random effects approach is therefore preferred because it retains the immigrant status coefficient while allowing unobserved time-invariant individual heterogeneity to be correlated with observed time-varying covariates.

The suitability of the correlated random effects formulation was further assessed by testing the joint significance of the person specific means of the time-varying covariates. These terms were jointly significant for both life satisfaction and mental health, supporting the use of the Mundlak specification rather than standard random effects. All main models were estimated with robust standard errors clustered at the individual level to account for heteroskedasticity and within person correlation across waves. Multicollinearity was assessed using variance inflation factors from pooled OLS approximations. The mean VIF was 1.55, and the VIF values for the substantive explanatory variables, including the social integration measures, were low. This suggests that multicollinearity is not a major concern. The results of these diagnostic checks are reported in Appendix B, Table B1.

The empirical analysis proceeds in stages. First, we estimate the raw subjective wellbeing gap between immigrants and natives. Second, we add demographic characteristics and economic integration controls. This provides the baseline model against which the contribution of social integration measures is assessed. Third, we introduce the social integration variables stepwise. This allows us to observe how the coefficient on immigrant status changes after the inclusion of British identity, community involvement, neighborhood attachment and UK-based social networks. A reduction in the immigrant coefficient after these variables are added is interpreted as evidence that differences in these dimensions account for part of the observed immigrant native subjective wellbeing gap.

It is important to clarify the interpretation of the estimates. The analysis is not designed to identify causal effects of social integration on subjective wellbeing. Although the correlated random effects approach helps account for time-invariant unobserved heterogeneity that is correlated with observed time-varying covariates, it does not remove all possible sources of endogeneity. In particular, the results may still be affected by reverse causality, omitted time varying factors, measurement error or unobserved processes that jointly shape subjective wellbeing and social integration. For example, individuals with higher subjective wellbeing may be more likely to participate in community life, form social ties or report stronger attachment to their neighborhood. Conversely, social integration may contribute to higher subjective wellbeing. These estimates should therefore be interpreted as conditional associations and as evidence on the extent to which measured differences in social integration account for the observed immigrant native subjective wellbeing gap, rather than as causal effects.

This interpretation is consistent with the purpose of the paper. The analysis asks whether differences in British identity, community involvement, neighborhood attachment and UK-based social networks help explain the remaining subjective wellbeing disparity between immigrants and natives after demographic and economic integration factors are taken into account. Future research could extend this analysis by using credible quasi experimental designs, stronger identification strategies or suitable instrumental variables where these are theoretically and empirically defensible. In the absence of such a design, the present paper offers panel-based evidence on the direction, strength and explanatory relevance of these associations within the UK context.

## 3 Results

This section first presents descriptive statistics for natives and immigrants, followed by the correlated random effects results for life satisfaction and mental health.

### 3.1 Descriptive statistics

Table 1 reports descriptive statistics for the dependent variables, demographic characteristics, economic integration controls and social integration measures separately for natives and immigrants. The analytical sample consists of 31,812 native observations and 5,731 immigrant observations across waves 3 and 6. Immigrants in the sample are slightly younger, healthier, more likely to be married, more highly educated and more likely to be employed than natives. However, they have lower levels of home ownership and lower mean scores on the principal social integration measures, including British identity, neighborhood attachment, UK-based social networks, volunteering and charitable behavior. These descriptive patterns motivate the regression analysis that follows, which examines whether such differences help account for immigrant native disparities in subjective wellbeing.

**Table 1:** Descriptive statistics for the dependent and independent variables.

Variables/category	Natives	Immigrants	Difference from natives
Demographic characteristics			
Female	55%	56%	
Relationship Status			
Unmarried	30%	25%	
Married	64%	69%	
Divorced	6%	6%	
Age mean	49	45	
Healthy	64%	73%	
Household size	2	4	
Economic integration controls			
Education			
No qualifications	11%	13%	
GCSE/Lower	30%	22%	
A-Level	22%	16%	
High Degree	37%	49%	
Employment status			
Employed	56%	59%	
Unemployed	4%	6%	
Outside labor market	40%	35%	
Net household monthly income	3,966	3,957	
Home ownership	74%	59%	
Social integration			
Neighborhood attachment	10.20	9.99	0.20^***^
Social networks	2.55	2.08	0.47^***^
Community involvement			
Volunteering	0.22	0.19	0.03^***^
Charitable behavior	0.75	0.66	0.09^***^
British identity	7.13	6.50	0.63^***^
Observations (waves 3,6)	31,812	5,731	
Number of Individuals	30,524	4,594	
Total observations (Overall sample)	37,543	
Total number of individuals (Overall sample)	35,118	

Total observations and number of individuals refer to the combined immigrant and native analytical sample. Differences from natives are reported where applicable. ^***^ denote statistical significance at the 1 percent levels, respectively.

### 3.2 Life satisfaction

Table 2 presents the results obtained from the correlated random effects estimation for differences in life satisfaction between immigrants and natives. For the sake of brevity and clarity, we do not report the means of time-varying variables as part of the regression table, although they are included in the model. The coefficient of the immigrant dummy can be interpreted as the gap in life satisfaction between immigrants and natives.

**Table 2:** Life satisfaction disparity between immigrants and natives (Correlated random effects model).

Variables/category		Life satisfaction (dependent variable)					
		Model 1a	Model 1b	Model 1c	Model 1d	Model 1e	Model 1f
Natives (ref.) Immigrants	−0.192^***^ (0.023)	−0.097^***^ (0.023)	−0.078^***^ (0.023)	−0.072^***^ (0.023)	−0.061^***^ (0.023)	−0.039^*^ (0.023)
Social integration							
British Identity				0.048^***^ (0.009)	0.048^***^ (0.009)	0.045^***^ (0.009)	0.045^***^ (0.009)
Volunteering					0.100^***^ (0.017)	0.071^***^ (0.017)	0.066^***^ (0.017)
Charitable behavior					0.088^***^ (0.018)	0.066^***^ (0.018)	0.060^***^ (0.018)
Neighborhood attachment						0.036^***^ (0.005)	0.036^***^ (0.005)
Social Network Size							0.030^**^ (0.014)
Demographic controls							
Male(ref.)	Female		0.069^***^ (0.015)	0.064^***^ (0.015)	0.056^***^ (0.015)	0.040^***^ (0.015)	0.037^**^ (0.015)
Age			−0.076^***^ (0.007)	−0.077^***^ (0.007)	−0.077^***^ (0.007)	−0.084^***^ (0.007)	−0.083^***^ (0.007)
Married (ref.)	Unmarried		−0.194^***^ (0.034)	−0.193^***^ (0.034)	−0.197^***^ (0.034)	−0.182^***^ (0.034)	−0.187^***^ (0.034)
	Divorced		−0.338^***^ (0.044)	−0.325^***^ (0.044)	−0.326^***^ (0.044)	−0.306^***^ (0.043)	−0.312^***^ (0.043)
Healthy			0.096^***^ (0.024)	0.100^***^ (0.023)	0.100^***^ (0.023)	0.101^***^ (0.023)	0.100^***^ (0.023)
Household Size			−0.034^**^ (0.016)	−0.036^**^ (0.016)	−0.035^**^ (0.016)	−0.035^**^ (0.016)	−0.035^**^ (0.016)
Economic integration controls							
Employed(ref.)	Unemployed		−0.371^***^ (0.045)	−0.372^***^ (0.045)	−0.363^***^ (0.045)	−0.361^***^ (0.044)	−0.355^***^ (0.044)
Outside the labor market			0.014 (0.035)	0.007 (0.035)	0.008 (0.035)	0.001 (0.034)	0.001 (0.034)
Home ownership			0.028 (0.032)	0.031 (0.032)	0.031 (0.032)	0.011 (0.032)	0.009 (0.032)
Educational Degree/High degree (ref.)	No qualifications		−0.079 (0.052)	−0.090^*^ (0.052)	−0.091^*^ (0.052)	−0.089^*^ (0.052)	−0.093^*^ (0.052)
GCSE/Lower			−0.126 (0.091)	−0.141 (0.091)	−0.138 (0.091)	−0.133 (0.090)	−0.136 (0.090)
A-levels			−0.136 (0.132)	−0.145 (0.132)	−0.140 (0.132)	−0.135 (0.131)	−0.133 (0.131)
Monthly household income			0.065^***^ (0.021)	0.066^***^ (0.020)	0.064^***^ (0.020)	0.065^***^ (0.020)	0.065^***^ (0.020)
Observations		37,543	37,543	37,543	37,543	37,543	37,543
Number of persons		35,118	35,118	35,118	35,118	35,118	35,118

All models include wave dummies. Models 1b to 1f include age squared, logged monthly household income and individual means of time-varying variables. Robust standard errors are reported in parentheses. ^***^, ^**^ and ^*^ denote statistical significance at the 1, 5 and 10 percent levels, respectively.

Looking first at the raw mean difference in Model 1a, immigrants report 0.19 points lower life satisfaction than natives. In Model 1b, after demographic characteristics and economic integration controls are included, this difference falls to 0.10 points. This indicates that these controls account for approximately half of the initial life satisfaction gap associated with immigrant status.

To show the substantive size of the adjusted life satisfaction gap, Model 1b can be compared with the coefficients for divorce and unemployment. Being divorced is associated with 0.33 points lower life satisfaction than being married, while being unemployed is associated with 0.37 points lower life satisfaction than being employed.

The 0.10-point lower life satisfaction reported by immigrants in Model 1b is therefore equivalent to approximately 30 percent of the estimated association between divorce and life satisfaction, and 27 percent of the estimated association between unemployment and life satisfaction. This suggests that the adjusted immigrant-native difference is substantively meaningful.

Model 1b is used as the baseline against which the role of social integration is assessed. When British identity is added in Model 1c, the immigrant coefficient falls from 0.10 to 0.08. This reduction indicates that differences in the reported importance of British identity account for part of the remaining life satisfaction gap after demographic and economic controls are included.

Model 1d adds the measures of community involvement, namely volunteering and charitable behavior. Both volunteering and charitable behavior are positively associated with life satisfaction, but their inclusion does not meaningfully reduce the immigrant coefficient further. This suggests that, although community involvement is positively associated with life satisfaction at the individual level, differences in volunteering and charitable behavior do not account for much of the immigrant-native life satisfaction gap in this sample. This indicates that not all dimensions of social integration contribute equally to explaining the observed disparity.

In Models 1e and 1f, neighborhood attachment and UK-based social networks are added to the specification, respectively. The inclusion of neighborhood attachment is associated with a further reduction in the immigrant coefficient, and the addition of social networks reduces it further. Comparing Models 1b to 1f, we observe that the significance of the initial gap in life satisfaction between immigrants and natives falls from 1 to 10%, while also reducing the magnitude of the immigrant dummy from 0.10 to 0.04. This suggests that the measured dimensions of social integration, particularly British identity, neighborhood attachment and UK-based social networks, account for a substantial share of the remaining life satisfaction gap between immigrants and natives after demographic and economic integration factors are considered.

The reduction in the immigrant coefficient is descriptive and should not be interpreted as evidence of a causal effect. It shows that measured differences in social integration are relevant to the observed disparity in life satisfaction.

In addition to the results for immigrant status, Table 2 shows the associations between the control variables and life satisfaction. Females and respondents without a long-standing illness or disability report higher life satisfaction, while being unmarried or divorced is negatively associated with life satisfaction. Age is negatively associated with life satisfaction and age squared is positive, indicating a non-linear pattern across the life course.

Turning to economic integration, unemployment is negatively associated with life satisfaction, while logged household income is positively associated with life satisfaction. Higher education and home ownership have positive but statistically insignificant associations in the fully specified models.

The social integration variables are generally positively associated with life satisfaction. British identity has a positive and statistically significant association, indicating that respondents who attach greater importance to being British report higher life satisfaction, net of the other covariates.

Volunteering and charitable behavior are also positively associated with life satisfaction. However, because these variables do not substantially reduce the immigrant coefficient, their role appears to be more relevant to overall life satisfaction than to explaining the immigrant native life satisfaction gap.

Finally, neighborhood attachment and UK-based social networks are positively associated with life satisfaction. These variables also reduce the immigrant coefficient, indicating that differences in local attachment and social networks are among the social dimensions most relevant to the observed life satisfaction disparity.

### 3.3 Mental health

Table 3 presents the results using mental health as the dependent variable. Model 2a shows that immigrants report a 0.16 point lower mental health score than natives. After demographic characteristics and economic integration controls are included in Model 2b, this difference falls to 0.11 points. This indicates that these controls account for part, but not all, of the observed mental health gap between immigrants and natives.

**Table 3:** Mental health disparity between immigrants and natives (Correlated random effects model).

Variables/category		Mental health (Dependent Variable)					
		Model 2a	Model 2b	Model 2c	Model 2d	Model 2e	Model 2f
Natives (ref.) Immigrants		−0.158^***^ (0.045)	−0.108^**^ (0.045)	−0.094^**^ (0.045)	−0.092^**^ (0.045)	−0.074^*^	−0.036 (0.046)
Social integration							
British identity				0.048^***^ (0.018)	0.048^***^ (0.018)	0.044^**^ (0.018)	0.044^**^ (0.018)
Volunteering					0.074^**^ (0.035)	0.029 (0.035)	0.020 (0.035)
Charitable behavior					0.005 (0.036)	−0.029 (0.035)	−0.040 (0.036)
Neighborhood attachment						0.062^***^ (0.010)	0.061^***^ (0.010)
Social Network Size							0.060^**^ (0.028)
Demographic controls							
Male(ref.)	Female		−0.391^***^ (0.029)	−0.394^***^ (0.029)	−0.396^***^ (0.029)	−0.421^***^ (0.029)	−0.427^***^ (0.029)
Age			−0.054^***^ (0.015)	−0.055^***^ (0.015)	−0.055^***^ (0.015)	−0.065^***^ (0.015)	−0.063^***^ (0.015)
Married (ref.)	Unmarried		−0.312^***^ (0.074)	−0.311^***^ (0.074)	−0.315^***^ (0.074)	−0.291^***^ (0.073)	−0.300^***^ (0.073)
Divorced			−0.178^*^ (0.093)	−0.168^*^ (0.093)	−0.170^*^ (0.093)	−0.139 (0.092)	−0.148 (0.092)
Healthy			0.321^***^ (0.049)	0.323^***^ (0.049)	0.323^***^ (0.049)	0.324^***^ (0.048)	0.323^***^ (0.048)
Household Size			−0.078^**^ (0.032)	−0.079^**^ (0.032)	−0.079^**^ (0.032)	−0.080^**^ (0.032)	−0.079^**^ (0.032)
Economic integration controls							
Employed (ref.)	Unemployed		−0.991^***^ (0.098)	−0.991^***^ (0.098)	−0.995^***^ (0.098)	−0.992^***^ (0.097)	−0.982^***^ (0.097)
Outside the labor market			−0.387^***^ (0.072)	−0.392^***^ (0.072)	−0.394^***^ (0.072)	−0.406^***^ (0.072)	−0.406^***^ (0.072)
Home ownership			−0.007 (0.064)	−0.004 (0.064)	−0.004 (0.064)	−0.035 (0.064)	−0.039 (0.064)
Educational Degree/High degree (ref.)	No qualifications		0.032 (0.105)	0.024 (0.105)	0.026 (0.105)	0.032 (0.104)	0.024 (0.104)
GCSE/Lower			−0.037 (0.185)	−0.046 (0.185)	−0.039 (0.185)	−0.029 (0.184)	−0.034 (0.184)
A-levels			−0.185 (0.270)	−0.188 (0.271)	−0.179 (0.271)	−0.167 (0.269)	−0.162 (0.269)
Monthly household income			0.121^***^ (0.040)	0.122^***^ (0.040)	0.122^***^ (0.040)	0.123^***^ (0.040)	0.123^***^ (0.040)
Observations		37,543	37,543	37,543	37,543	37,543	37,543
Number of persons		35,118	35,118	35,118	35,118	35,118	35,118

All models include wave dummies. Models 2b to 2f include age squared, logged monthly household income and individual means of time-varying variables. Robust standard errors are reported in parentheses. ^***^, ^**^ and ^*^ denote statistical significance at the 1, 5 and 10 percent levels, respectively.

To communicate the substantive size of the mental health gap, we again compare the immigrant coefficient with the estimates for divorce and unemployment. In Model 2b, being divorced is associated with 0.18 points lower mental wellbeing compared to being married, while unemployment is associated with a 0.99 points lower mental wellbeing compared with employment. The 0.11-point mental health gap between immigrants and natives in Model 2b is therefore equivalent to approximately 61 percent of the estimated association between divorce and mental wellbeing, and 11 percent of the estimated association between unemployment and mental wellbeing. This comparison suggests that the immigrant-native mental health gap is meaningful, although smaller than the estimated association for unemployment.

The introduction of social integration measures follows the same stepwise logic as in the life satisfaction analysis. In Model 2c, the inclusion of British identity reduces the immigrant coefficient, suggesting that differences in British identity account for part of the mental health gap that remains after demographic and economic controls. In Model 2d, the inclusion of community involvement does not meaningfully reduce the immigrant coefficient. As in the life satisfaction analysis, this indicates that volunteering and charitable behavior are not the main dimensions accounting for the immigrant-native mental health gap in this sample.

A further reduction in the immigrant coefficient is observed when neighborhood attachment and UK-based social networks are introduced in Models 2e and 2f. In the final model, the immigrant coefficient becomes statistically insignificant. This indicates that the measured differences in British identity, neighborhood attachment and UK-based social networks account for much of the remaining mental health gap between immigrants and natives.

The results further show that British identity, neighborhood attachment and UK-based social networks are positively associated with mental health. Volunteering is positive in some specifications, while charitable behavior is not statistically significant in the fully specified model.

The findings for mental health broadly reinforce those obtained for life satisfaction. In both cases, demographic characteristics and economic integration explain part of the immigrant-native gap, and the inclusion of social integration measures accounts for a further share of the remaining disparity.

Taken together, the results show that the explanatory relevance of social integration is uneven across dimensions. British identity, neighborhood attachment and UK-based social networks have the clearest role in accounting for the immigrant native subjective wellbeing gap, while community involvement plays a more limited role.

## 4 Discussion and conclusion

This paper examined whether immigrants in the UK report lower subjective wellbeing than natives, and whether British identity, community involvement, neighborhood attachment and UK-based social networks help account for this disparity after demographic characteristics and economic integration factors are taken into account. The results show that immigrants report lower life satisfaction and mental health than natives. This gap is reduced after including demographic and economic controls, but it is not fully removed. The inclusion of social integration measures accounts for a further share of the remaining gap, especially through British identity, neighborhood attachment and UK-based social networks. Community involvement, measured through volunteering and charitable behavior, has a more limited explanatory role.

These findings reinforce a central point in the recent literature: immigrant-native differences in subjective wellbeing cannot be understood through economic integration alone. They are consistent with studies showing persistent wellbeing differences between immigrants and natives in Europe and with work emphasizing the relevance of discrimination, social capital and social embeddedness for immigrant wellbeing (Safi, 2010; Kirmanoglu and Başlevent, 2014; De Vroome and Hooghe, 2014; Tegegne and Glanville, 2019; Zafar and Ammara, 2023). At the same time, the results do not diminish the importance of economic integration. Employment, income, education and home ownership remain important controls because they capture material security, labor market position and settlement resources, as shown in previous work on immigrant life satisfaction, unemployment and home ownership (Bartram, 2011; Kóczán, 2016; Leopold et al., 2017; Shen and Kogan, 2020; Son and Choi, 2026).

The clearest explanatory dimensions in this paper are British identity, neighborhood attachment and UK-based social networks. The association between British identity and subjective wellbeing is consistent with social identity perspectives, which emphasize the role of group belonging, recognition and identity in wellbeing (Tajfel and Turner, 1986; Jetten et al., 2012). It also aligns with empirical work showing that host country identification and British national identification are associated with life satisfaction (Manning and Roy, 2010; Casey and Dustmann, 2009; Angelini et al., 2015; Jaspal et al., 2021). In the present paper, this should not be read as an argument for assimilation or the abandonment of origin identities. Rather, British identity is interpreted as one observable indicator of subjective belonging within the host society.

The findings on neighborhood attachment and UK-based social networks also sit well with studies emphasizing the importance of local embeddedness, social contact and informal social capital. Appau et al. (2019) show that neighborhood belonging and interaction are associated with life satisfaction in the UK general population, while Knies et al. (2016) show that neighborhood context matters for life satisfaction among ethnic minority groups in England. Ambrosetti and Paparusso (2024) similarly find that subjective neighborhood quality is positively associated with life satisfaction among immigrants and natives in Europe. The role of UK-based social networks is consistent with (Arpino and de Valk 2018), who show that social contacts explain part of the immigrant-native life satisfaction gap, and with Tegegne and Glanville (2019), who find that informal social connections and trust account for a substantial share of the non-western immigrant-native wellbeing gap. The UK qualitative evidence of Wessendorf and Phillimore (2019) is also relevant here because it shows that migrant settlement is supported by a range of social relations, including acquaintances, co-ethnic ties, other migrant networks and majority-group contacts. This supports the interpretation of UK-based friendships as a practical and emotional settlement resource rather than as a narrow measure of majority-group bridging only.

The more limited explanatory role of community involvement is also informative. Volunteering and charitable behavior are positively associated with some wellbeing outcomes, but they do not account for much of the immigrant-native gap once other dimensions of social integration are included. This finding should be read alongside studies that link volunteering, charitable giving and community participation to integration and wellbeing (Andreoni, 1990; Osili and Du, 2005; Handy and Greenspan, 2009; Baert and Vujić, 2016; Greenspan et al., 2018; Appau et al., 2019). It is also consistent with the mixed evidence from immigrant-specific studies. Ramos et al. (2017) and Hombrados-Mendieta et al. (2013) find positive links between community involvement, sense of community and life satisfaction, while Miller et al. (2020) show that formal volunteering is not significantly associated with migrant mental wellbeing in Tokyo once other factors are considered. The present findings therefore suggest that formal civic participation is not interchangeable with belonging, local attachment or close interpersonal networks.

The mental health results extend this interpretation beyond life satisfaction. The disappearance of the immigrant coefficient in the fully specified mental health model suggests that the measured social integration dimensions are particularly relevant to psychological wellbeing. This is consistent with Brydsten et al. (2019), who show that social integration, social support, trust and economic insecurity explain part of mental health inequalities between foreign-born and native-born people in Sweden. It also accords with wider evidence that immigrant mental health is shaped by post-migration stressors, social support and the broader conditions of settlement (Jayaweera, 2014; Gilliver et al., 2014; Hou et al., 2020).

The contribution of this paper is therefore not that social integration has been entirely absent from previous research. Rather, the contribution is to examine several theoretically grounded dimensions of social integration jointly within a UK immigrant-native panel framework, and to ask which of these dimensions helps account for the remaining wellbeing gap after economic integration factors are included. This responds directly to the fragmented nature of the existing literature, where identity, neighborhood context, social capital, community involvement and mental health are often examined in separate studies, in immigrant-only samples, general population samples or non-UK contexts.

The findings also have implications for integration policy. Policies concerned with immigrant integration often focus on employment, income, education, language and housing, reflecting the importance of structural integration (Ager and Strang, 2008; Saggar et al., 2012; Paparusso, 2021; OECD and European Commission, 2023). These remain essential. However, the results suggest that an exclusive focus on economic outcomes provides an incomplete account of immigrant wellbeing. If differences in subjective wellbeing are also associated with British identity, neighborhood attachment and UK-based social networks, then integration policy should also consider the conditions that support social connection, local embeddedness and recognized belonging. This does not mean that belonging can be imposed, or that integration should be understood narrowly as assimilation. It means that opportunities for everyday contact, local participation, neighborly support and meaningful social ties are relevant to the wellbeing dimension of integration.

The findings should be interpreted with caution. The correlated random effects approach accounts more carefully for time-invariant unobserved individual heterogeneity than a single cross-section and retains the immigrant status coefficient, but it does not establish causality. The associations may be shaped by reverse causality, omitted time-varying factors, measurement error or unobserved processes that jointly influence subjective wellbeing and social integration. For example, individuals with higher wellbeing may be more likely to develop social ties, participate in community life or report stronger neighborhood attachment. Conversely, social integration may contribute to higher wellbeing. The results should therefore be interpreted as evidence that measured social integration differences help account for the observed immigrant-native wellbeing gap, not as causal estimates of the effects of social integration.

The measures also have limitations. British identity is measured with a single item and should not be interpreted as a complete measure of bicultural identity or acculturation. UK-based friendship captures whether close friends are located in the UK, but not the quality, frequency, ethnic composition or intensity of those relationships. Neighborhood attachment captures local interaction, advice and reciprocal exchange, but not all features of neighborhood quality. Community involvement is measured through volunteering and charitable behavior, and the timing of these variables requires a data harmonization strategy. The robustness checks reported in Appendix A indicate that the main conclusions are not driven by this treatment, but the community involvement results should still be interpreted cautiously.

Future research could extend the analysis in several directions. First, it could examine heterogeneity by immigrant generation, origin region, length of residence, legal status or reason for migration. Second, it could examine whether the associations between social integration and subjective wellbeing differ between immigrants and natives through interaction models. Third, future studies could incorporate additional dimensions of integration, including discrimination, religion, language, citizenship and the ethnic composition of social networks. Finally, stronger causal designs would be valuable where credible instruments, policy changes or quasi-experimental settings are available. A further limitation is that panel attrition may affect the analytical sample if immigrants who leave the survey differ systematically from those who remain in their wellbeing, settlement experiences or social integration.

Overall, the paper shows that the immigrant-native subjective wellbeing gap in the UK is not only an economic question. Demographic and economic characteristics explain part of the gap, but social integration also matters. British identity, neighborhood attachment and UK-based social networks are particularly relevant in accounting for the remaining disparity, while community involvement has a more limited role. These findings point to the importance of understanding integration as a multidimensional process involving economic security, social connection, local belonging and subjective recognition within the host society.

## Data Availability

Publicly available datasets were analyzed in this study. This data can be found here: DOI: http://doi.org/10.5255/UKDA-SN-6614-21.

## Footnotes

1 This question is not asked of people who do not have friends, and these respondents are therefore coded in the ‘0' category.
