# Supplementary material for: The wellbeing gap between immigrants and natives: the role of social integration
Source: Front Sociol. 2026 Jun 19;11:1815828. doi: 10.3389/fsoc.2026.1815828 (PMC13330107; doi:10.3389/fsoc.2026.1815828)
Supplement: Supplementary file 1 [file Supplementary_file_1.pdf]

Appendix A: Data Harmonisation and Robustness checks for Community Involvement

Table A1. Life satisfaction disparity between immigrants and natives excluding community involvement

		Life Satisfaction (Dependent Variable)				
		Model 3a	Model 3b	Model 3c	Model 3d	Model 3e
**Immigrants**	Native (ref.)	-0.192***	-0.096***	-0.078***	-0.065***	-0.041*
		(0.023)	(0.023)	(0.023)	(0.023)	(0.023)
**Social Integration**						
	British Identity			0.048***	0.045***	0.045***
				(0.009)	(0.009)	(0.009)
	Neighbourhood attachment				0.036***	0.036***
					(0.005)	(0.005)
	Social Network Size					0.033**
						(0.014)
Demographic controls		No	Yes	Yes	Yes	Yes
Economic integration controls		No	Yes	Yes	Yes	Yes
Means of time-varying variables		No	Yes	Yes	Yes	Yes
Wave Dummies		Yes	Yes	Yes	Yes	Yes
Observations		37,543	37,543	37,543	37,543	37,543
Number of persons		35,118	35,118	35,118	35,118	35,118

Robust standard errors in parentheses ***, **, * denote statistical significance at the 1, 5, and 10 percent levels, respectively

Table A2. Life satisfaction disparity between immigrants and natives (wave 6 only cross-sectional model)

		Life Satisfaction (Dependent Variable)					
		Model 4a	Model 4b	Model 4c	Model 4d	Model 4e	Model 4f
**Immigrants**	Native (ref.)	-0.145***	-0.129***	-0.107***	-0.100***	-0.094***	-0.072**
		(0.025)	(0.025)	(0.025)	(0.025)	(0.025)	(0.025)
**Social Integration**							
	British Identity			0.041***	0.041***	0.038***	0.037***
				(0.003)	(0.003)	(0.003)	(0.003)
	Volunteering				0.124***	0.100***	0.093***
					(0.018)	(0.018)	(0.018)
	Charitable behaviour				0.099***	0.082***	0.073***
					(0.019)	(0.019)	(0.019)
	Neighbourhood attachment					0.059***	0.057***
						(0.003)	(0.003)
	Social Network Size						0.064***
							(0.010)
Demographic controls		No	Yes	Yes	Yes	Yes	Yes
Economic integration controls		No	Yes	Yes	Yes	Yes	Yes
Means of time-varying variables		No	Yes	Yes	Yes	Yes	Yes
Wave Dummies		Yes	Yes	Yes	Yes	Yes	Yes
Observations		33,884	33,884	33,884	33,884	33,884	33,884
Number of persons		33,884	33,884	33,884	33,884	33,884	33,884

Robust standard errors in parentheses ***, **, * denote statistical significance at the 1, 5, and 10 percent levels, respectively.

Table A3. Mental health disparity between immigrants and natives excluding community involvement

		Mental health (Dependent Variable)				
		Model 5a	Model 5b	Model 5c	Model 5d	Model 5e
**Immigrants**	Native (ref.)	-0.158***	-0.101**	-0.087**	-0.068*	-0.030
		(0.045)	(0.045)	(0.045)	(0.045)	(0.046)
**Social Integration**						
	British Identity			0.047***	0.043**	0.043**
				(0.018)	(0.018)	(0.018)
	Neighbourhood attachment				0.062***	0.061***
					(0.010)	(0.010)
	Social Network Size					0.061**
						(0.028)
Demographic controls		No	Yes	Yes	Yes	Yes
Economic integration controls		No	Yes	Yes	Yes	Yes
Means of time-varying variables		No	Yes	Yes	Yes	Yes
Wave Dummies		Yes	Yes	Yes	Yes	Yes
Observations		37,543	37,543	37,543	37,543	37,543
Number of persons		35,118	35,118	35,118	35,118	35,118

Robust standard errors in parentheses ***, **, * denote statistical significance at the 1, 5, and 10 percent levels, respectively

Table A4. Mental health disparity between immigrants and natives (wave 6 only cross-sectional model)

		Mental Health (Dependent Variable)					
		Model 6a	Model 6b	Model 6c	Model 6d	Model 6e	Model 6f
**Immigrants**	Native (ref.)	-0.128***	-0.125**	-0.106**	-0.104**	-0.095*	-0.051
		(0.050)	(0.049)	(0.049)	(0.049)	(0.049)	(0.049)
**Social Integration**							
	British Identity			0.038***	0.038***	0.033***	0.031***
				(0.005)	(0.005)	(0.005)	(0.005)
	Volunteering				0.075**	0.035	0.021
					(0.037)	(0.037)	(0.037)
	Charitable behaviour				0.009	-0.019	-0.037
					(0.038)	(0.038)	(0.038)
	Neighbourhood attachment					0.095***	0.091***
						(0.006)	(0.006)
	Social Network Size						0.124***
							(0.020)
Demographic controls		No	Yes	Yes	Yes	Yes	Yes
Economic integration controls		No	Yes	Yes	Yes	Yes	Yes
Means of time-varying variables		No	Yes	Yes	Yes	Yes	Yes
Wave Dummies		Yes	Yes	Yes	Yes	Yes	Yes
Observations		33,884	33,884	33,884	33,884	33,884	33,884
Number of persons		33,884	33,884	33,884	33,884	33,884	33,884

Robust standard errors in parentheses ***, **, * denote statistical significance at the 1, 5, and 10 percent levels, respectively.

Table A5. Stability of volunteering and charitable behaviour across waves 4 and 6

	Wave 4 mean		Wave 6 mean		Same category, waves 4 to 6	Correlation, waves 4 to 6
**Measure**	**Natives**	**Immigrants**	**Natives**	**Immigrants**		
Volunteering	0.207	0.174	0.213	0.189	82.9 per cent	0.476
Charitable Behaviour	0.712	0.641	0.750	0.685	75.8 per cent	0.392

Appendix B: Model evaluation and diagnostic checks

Table B1. Model evaluation and diagnostic checks results

Diagnostic check	Life satisfaction	Mental health
Breusch Pagan LM test for random effects	$\chi^2 = 241.23$, $p < 0.001$	$\chi^2 = 330.77$, $p < 0.001$
Hausman test	$\chi^2 = 89.70$, $p < 0.001$	$\chi^2 = 50.98$, $p < 0.001$
Joint test of Mundlak terms	$\chi^2 = 613.90$, $p < 0.001$	$\chi^2 = 769.47$, $p < 0.001$
Joint test of social integration variables	$\chi^2 = 104.72$, $p < 0.001$	$\chi^2 = 44.08$, $p < 0.001$
Wald chi square, final CRE model	$\chi^2 = 3967.79$, $p < 0.001$	$\chi^2 = 3547.52$, $p < 0.001$
Overall R squared, final CRE model	0.103	0.113
Within R squared, final CRE model	0.008	0.021
Between R squared, final CRE model	0.105	0.114
Observations	37,543	37,543
Individuals	35,118	35,118
Mean VIF	1.55	1.55
Clustered robust standard errors	Yes	Yes
